# In defense of adolescents: They really do use braces for the hours prescribed, if good help is provided. Results from a prospective everyday clinic cohort using thermobrace

**DOI:** 10.1186/1748-7161-7-12

**Published:** 2012-05-31

**Authors:** Sabrina Donzelli, Fabio Zaina, Stefano Negrini

**Affiliations:** 1 ISICO (Italian Scientific Spine Institute), Via Bellarmino 13/1, 20141, Milan, Italy; 2 Physical and Rehabilitation Medicine, University of Brescia, Brescia, Italy; 3 Care & Research Institute Don Gnocchi Foundation, Milan, Italy

## Abstract

**Background:**

The effectiveness of bracing relies on the quality of the brace, compliance of the patient, and some disease factors. Patients and parents tend to overestimate adherence, so an objective assessment of compliance has been developed through the use of heat sensors. In 2010 we started the everyday clinical use of a temperature sensor, and the aim of this study is to present our initial results.

**Methods:**

Population: A prospective cohort of 68 scoliosis patients that finished at least 4 months of brace treatment on March 31, 2011: 48 at their first evaluation (79% females, age 14.2±2.4) and 20 already in treatment.

Treatment: Bracing (SPoRT concept); physiotherapic specific exercises (SEAS School); team approach according to the SOSORT Bracing Management Guidelines.

Methods. A heat sensor, “Thermobrace” (TB), has been validated and applied to the brace. The real (measured by TB) and referred (reported by the patient) compliances were calculated.

Statistics. The distribution was not normal, hence median and 95% interval confidence (IC95) and non-parametric tests had to be used.

**Results:**

Average TB use: 5.5±1.5 months. Brace prescription was 23 hours/day (h/d) (IC95 18–23), with a referred compliance of 100% (IC95 70.7-100%) and a real one of 91.7% (IC95 56.6-101.7%), corresponding to 20 h/d (IC95 11–23). The more the brace was prescribed, the more compliant the patient was (94.8% in 23 h/d vs. 73.2% in 18 h/d, P < 0.05). Sixty percent of the patients had at least 90% compliance, and 45% remained within 1 hour of what had been prescribed. Non-wearing days were 0 (IC95 0–12.95), and involved 29% of patients.

**Conclusion:**

This is the first study using a TB in a setting of respect for the SOSORT criteria for bracing, and it states that it is possible to achieve a very good compliance, even with a full time prescription, and better than what was previously reported (80% maximum). We hypothesize that the treating team (SOSORT criteria) plays a major role in our results. This study suggests that compliance is neither due to the type of treatment only nor to the patient alone. According to our experience, TB offers valuable insights and do not undermine the relationship with the patients.

## Introduction

The efficacy of bracing in the treatment of adolescent idiopathic scoliosis is controversial. The recently published Cochrane review [1] stated that there is very low-quality evidence in favor of the brace, suggesting that patients’ choices should be informed in multidisciplinary discussion and that compliance and the standard of bracing must be considered. Some authors reported the control of curve progression with bracing [2-10], while a review by Dolan of the English literature, published in 2007 [11], found no advantage of bracing over observation in terms of surgical rate. However, the author elected to exclude from the review the studies with the combined approach of bracing and exercises, where the results appear to be good [1,6,7].

Compliance is a key element in the efficacy of brace treatment, accompanied by other variables: brace efficacy, but also spinal flexibility, curve type and magnitude, skeletal maturity, and others [12-14]. Compliance is one of the key elements behind the Recommendations on Bracing Management established by the Society on Scoliosis Orthopedic and Rehabilitation Treatment (SOSORT) in 2008 [15], as reported also in the 2011 SOSORT Guidelines: Orthopaedic and Rehabilitation Treatment of Idiopathic Scoliosis During Growth [16]. According to some published results, in patients at risk it is possible to avoid surgery through bracing: by respecting the SOSORT criteria and focusing on compliance, a complete, conservative treatment based on bracing and exercises produced results, according to the SRS criteria, far exceeding what has been reported previously [3-5,8,9]. Compliance could well be one of the main reasons for the differences of efficacy reported in the literature.

Previous studies have used either questionnaires or verbal reports to determine compliance [17,18], but the number of hours reported was subjective and difficult to verify. A study by Morton [19] demonstrated, by comparing the prediction and estimation of adherence to the objective data on compliance monitoring, that the prediction of future adherence to brace treatment is difficult and error-prone. The unreliability of estimates of compliance with brace treatment brings into question the validity of unmonitored studies of brace treatment for scoliosis. Consequently, several groups have developed objective compliance measures [20-27]. The measures of compliance reported using different types of data loggers, which varied among the studies from 65% to 78%, [12,21-27] with studies documenting compliance as low as 33% [20] and 47% [19].

Some authors published results concerning the relationship between the measured number of hours of brace wear and the control of curve progression. Katz [26] showed that the patients with the highest percentage of compliance to the hours prescribed had less curve progression. Takemitsu and Rahman [23] stated that patients with greater than 90% compliance were five times more likely to have a favorable outcome than those with less than 90%.

Considering what has been found in previous studies, we started an everyday application in our clinical setting with full respect for the SOSORT criteria for bracing management [15] of an objective brace temperature measurement system we called “Thermobrace” (TB). The objective of the study is to verify whether, in specific settings that respect the SOSORT criteria [15], it is possible to achieve good compliance close to that declared by the patients. The secondary objective is to assess whether the use of this device could be useful not only for research but also for everyday clinical practice.

## Material and methods

### Design

This is a prospective cohort observational uncontrolled study focusing on everyday clinical use of a heat sensor (TB) to check compliance in patients braced for spinal deformities.

### Methods

At brace prescription, if the physicians deemed it to be appropriate during the consultation, the patients’ families have been asked whether they wanted to check the compliance of their children to treatment through TB for clinical purposes: If this was the case, they had to buy TB, thus confirming their adherence to the project. TB was then applied by the orthotist during brace construction and the data was read by the treating physician at the next clinical evaluation. All parents of participating patients signed a consent form for clinical research. Because it was our institute’s first experience with the clinical introduction of the TB, some caution was applied at the beginning by the prescribing physicians, primarily for the purpose of avoiding any interference with a good physician-patient-family relationship. [27] Consequently, at the start it was proposed as complimentary (i.e. the patients and family had to decide if they want to buy it), and mainly to patients already in treatment who were showing problems in the use of the brace. Subsequently, it was also proposed as complimentary to the patients at their first evaluation at our institute, but not to all of them. At that time we did not have a real rule for inclusion/exclusion of the patients: exclusion were done only basing on factors like difficulties of patient/family/physician relationship of any kind; delays that precluded to have time to discuss also the choice of using TB or not; families who were arbitrarily thought that could have had economic problems; and so on. Only recently, following the results of this study and the clinical perception of its utility, it has become a standard clinical procedure in our institute (i.e., prescribed to all patients). Consequently, the prescription of the TB gradually increased with time, but with caution until the first TB results were received, in order to have time to check them as well as the possible consequent difficulties in managing patients. After this test period we began to understand that the regular use of this device should enrich our activity: that’s why we start the standard prescription of Thermobrace.

### Patients

We report here on all the first patients who consecutively accepted the voluntary use of the TB for the clinical evaluation of brace usage. Sixty-eight adolescent idiopathic scoliosis (93%) or hyperkyphosis (7%) patients with brace prescriptions were recruited during our everyday clinical activity. There were 53 females, 15 males; the mean age was 14 years 6 months ± 2 years 3 months; scoliosis patients had maximum curvature of 41.4 ± 10.5 Cobb degrees at the start of treatment. In Table 1 the characteristics of these sub-groups are reported. All the study has been carried out with respect to the first group, while the second was used only to check whether referred compliance changed after starting the use of the TB.

**Table 1:** Characteristic of the two studied groups

		**Patients at first brace prescription**	**Patients already braced**	**P**
*Number*		48	20	-
*Age*		14.06 ± 1.12	14.06 ± 2.09	NS
*Gender*	*Males*	10	5	NS
	*Females*	38	15	
*Cobb pre-bracing*	*Average*	36.8 ± 10.6	32.7 ± 10.4	NA
	*Proximal thoracic*	28.5 ± 12.1	19.0*	
	*Thoracic*	40.3 ± 10.3	36.3 ± 16.5	
	*Thoraco-lumbar*	36.2 ± 11.2	31.7 ± 6.7	
	*Lumbar*	30.7 ± 14.2	44.0 ± 19.7	
*Cobb at evaluation*	*Average*	30.3 ± 4.0	31.6 ± 15.2	NA
	*Proximal thoracic*	23.8 ± 7.3	19.3*	
	*Thoracic*	34.0 ± 12.6	34.4 ± 15.7	
	*Thoraco-lumbar*	30.6 ± 9.8	23.0 ± 10.8	
	*Lumbar*	30.1 ± 18.2	36.4 ± 15.3	
*Risser test pre-bracing*		1.9 ± 1.9	0 ± 1.22	<0.05
*Risser test at start of this study*		2.6 ± 1.3	1.2 ± 1.5	NS
*Curve type*	*Single thoracic*	11	1	NA
	*Single thoracolumbar*	2	0	
	*Single lumbar*	2	1	
	*Double thoracic-lumbar*	17	8	
	*Moe*	8	6	
	*Others*	8	4	
*Brace prescribed*	*Sforzesco*	42	16	NS
	*Sibilla*	3	4	
	*Lapadula*	3	0	
*Hours prescribed*	*23 h/day*	35	7	<0.05
	*18*–*22 h/day*	7	4	
	*8*–*17 h/day*	7	10	

Characteristic of the two studied groups. NS: not significant; NA: not applicable. *: only one patient.

Since this is an observational study of clinical everyday behaviors, it did not require approval of an ethics committee. All patients included in this study signed an informed consent to clinical data management for research purposes.

### Temperature data monitor

**Figure 1:**
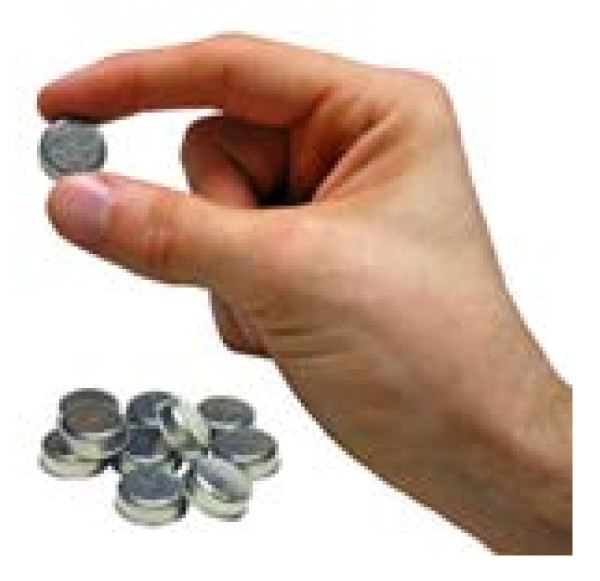
**The heat-sensor device used in this study: “Thermobrace”.** The heat-sensor device used in this study, “iButton™ DS1922L-F5#” (http://www.maxim-ic.com/datasheet/index.mvp/id/4088/t/al) (Maxim Integrated Products, Inc.; 120 San Gabriel Drive Sunnyvale, CA 94086), which we called “Thermobrace” for this specific use.

**Figure 2:**
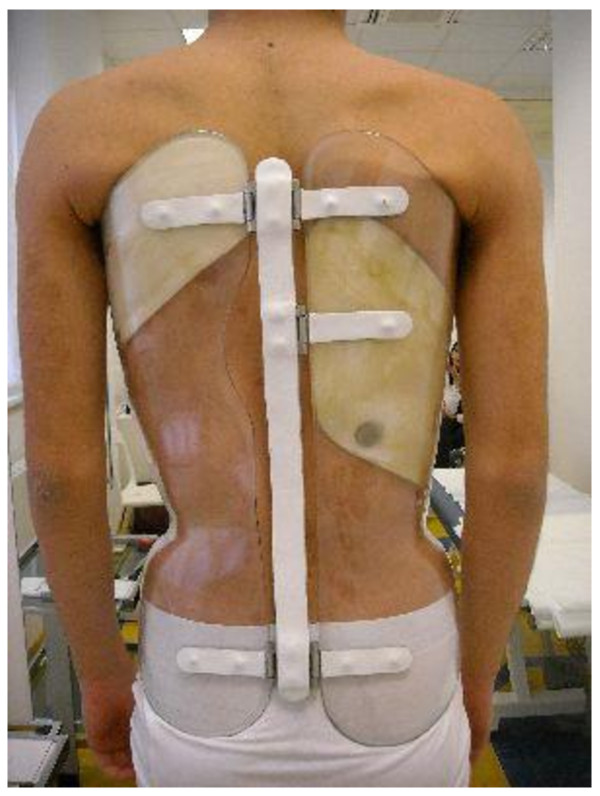
**Placement of the Thermobrace in a Sforzesco brace.** Example of placement of the Thermobrace in a Sforzesco brace worn by a patient included in the study.

To measure the actual brace wear, we used a commercially available heat-sensor device called the “iButton™ DS1922L-F5#” (http://www.maxim-ic.com/datasheet/index.mvp/id/4088/t/al) (Maxim Integrated Products, Inc.; 120 San Gabriel Drive, Sunnyvale, CA 94086), which we called for this specific use “Thermobrace” (Figure 1). It is a small temperature data logger to be installed within each orthosis (Figure 2) constituted by a heat sensor, a battery and a memory. The sensor is meant to measure the brace temperature, and it is not placed in contact with the patient but within a pressure pad in the brace. Recently a reliability study has been published on this specific instrument [28].

### Preliminary validation trial

Before starting the clinical application we performed a preliminary validation trial involving 5 female patients: They all had adolescent idiopathic scoliosis (range 35-53° Cobb), were recruited on a voluntary basis, and had been in brace treatment for more than 2 years. Two of them were already known for having bad referred compliance, while 3 had very good referred compliance. Since before this study we were not using any monitoring device, the only type of compliance we already knew of these patients was what they referred when inquired in front of their parents. Referred compliance has been shown to underestimate the real brace wearing [19].

We required all these patients to register on a data sheet each time they put on the brace or took it off, and we compared this data with that registered by the TB. We considered their data sheets as the golden standard reference to validate TB measurements. In fact, the data sheets were completely different from referred compliance: pupils had to fill in them during the day reporting exactly not the time of brace wearing, but the hour and minutes of the day so that calculation could be made by us afterwards. Moreover, patients did know that what we were searching for was the validity of the instrument and not their own compliance. Parents were asked not to check this data sheet, and we guaranteed we would have not used them to check their compliance (what we did, in fact). Finally, they had to perform this validation for one month, while the period of observation for their treatment was six months (so detaching compliance to therapy from the observation period of the pilot study).

The preliminary trial lasted 27.5 days (full memory): The TB showed good precision, with a difference between real and registered data ranging from 0 to 7.7% (Table 2). The two patients with the worst results were the non-compliant and had frequent brace on/off cycles. However, when the brace was worn regularly the reliability of the TB was very good (range 0–0.4% in compliant).

**Table 2:** Results of the preliminary validation trial

		**Questionnaire**		**Thermobrace**		**Difference**	
		**Hours of bracing registered**		**Hours of bracing calculated**		**Hours**	
		**Total**	**Per day**	**Total**	**Per day**	**Total**	**%**
*Patient 1*	*compliant*	599	21.8	597	21.7	2	0.40
*Patient 2*	*compliant*	444	16.3	444	16.3	0	0
*Patient 3*	*non-compliant*	320	11.8	277	10.2	42	6.49
*Patient 4*	*compliant*	426	15.5	429	15.6	3	0.40
*Patient 5*	*non-compliant*	531	19.4	480	17.5	51	7.70

Data from the preliminary trial that showed good precision of the heat sensor used.

In the choice of the final time-span between each single measurement, we had to make a compromise. In fact, ideally the best solution would have been to measure temperature every minute, but there were memory limitation for an everyday clinical usage where patients are usually seen every 4 to 6 months (with some of them, usually low compliant, not coming back before 8–10 months). In fact, the more memory we used, the higher the costs of the device, and to let patients freely buy it we had to maintain this costs below 80–100 Euros, a threshold that was established after a preliminary informal inquiry with a number of patients. As a consequence, based on the recorded data (every 20 minutes) we checked the minimum time-span in order to discern the real use of the brace without losing precision between two clinical evaluations. After this trial we concluded that the TB was reliable and it was possible to sample the temperature every 60 minutes, thus optimizing the memory with good-quality data. It was also decided to develop a reliability test for clinical purposes, as explained below.

### Reading software

A specific software program with which to elaborate the data has been developed and is now freely available on-line (http://www.scoliosismanager.org/thermobrace). To determine whether a sample corresponds to the brace being worn/not worn, it uses the following algorithm:

1) Evaluating all the temperature samples collected, AvMax and AvMin are defined as the average value of the 100 highest and lowest samples

2) The average Av is defined as the average between AvMax and AvMin;

3) The monthly moving average AvHigh_t_ is defined as the average of values over Av from 15 days before to 15 days after sample “t” (i.e., the actual temperature that we are interested in defining as “brace worn” or “brace not-worn”); moving average values are necessary to take into account global temperature variations due to seasons (winter, summer, etc.)

4) The moving average AvLowt is defined as the average of values under Av from 15 days before to 15 days after sample “t”

After the calculation of those parameters, the samples are evaluated with consideration for the following:

The samples over AvHigh_t_: brace worn

The samples below AvLow_t_: brace not worn

For the other samples: Looking at previous and subsequent samples, evaluation of the phase (brace dressing/undressing) to assign the value worn/not worn.

This method is sometimes not reliable with patients wearing braces 23 h/d. If the sampling period is too short (less than 3 months) or if the patient wears the brace all day with the exception of 20–30 minutes a day (which some of them do), the lower samples are not sufficient to reliably define the thresholds. This is why we defined the reliability index, to make the physician aware of potential problems. The reliability index evaluates many parameters, like the number of transitions brace on/brace off; degrees of interval between high threshold (brace certainly worn) and low threshold (brace certainly not worn); and number of samples. The index defines the reliability of the calculation of the threshold between brace worn/not worn (e.g., a patient who puts on or removes his/her brace many times during the day reduces the reliability).

Moreover, we defined another experimental calculation method: after collecting data of more than 50 patients, in different seasons (winter, summer), they were evaluated to find the mean thresholds of brace worn/not worn in their samples. We identified the thresholds as 28° (brace worn) and 25° (brace not worn). This second method simply assigned the samples with respect to those values.

In this specific study the data reliability was 84.0% (IC95 46.1-100) for the first of the two processing methods, versus 82.2% (IC95 39.4-100) for the second, thus indicating a statistically significant difference. Only 10 patients had better reliability results with the second method. In this study we consider only the most reliable data-processing method obtained in each single case (reliability: 84.3%; IC 95 46.1-100).

**Figure 3:**
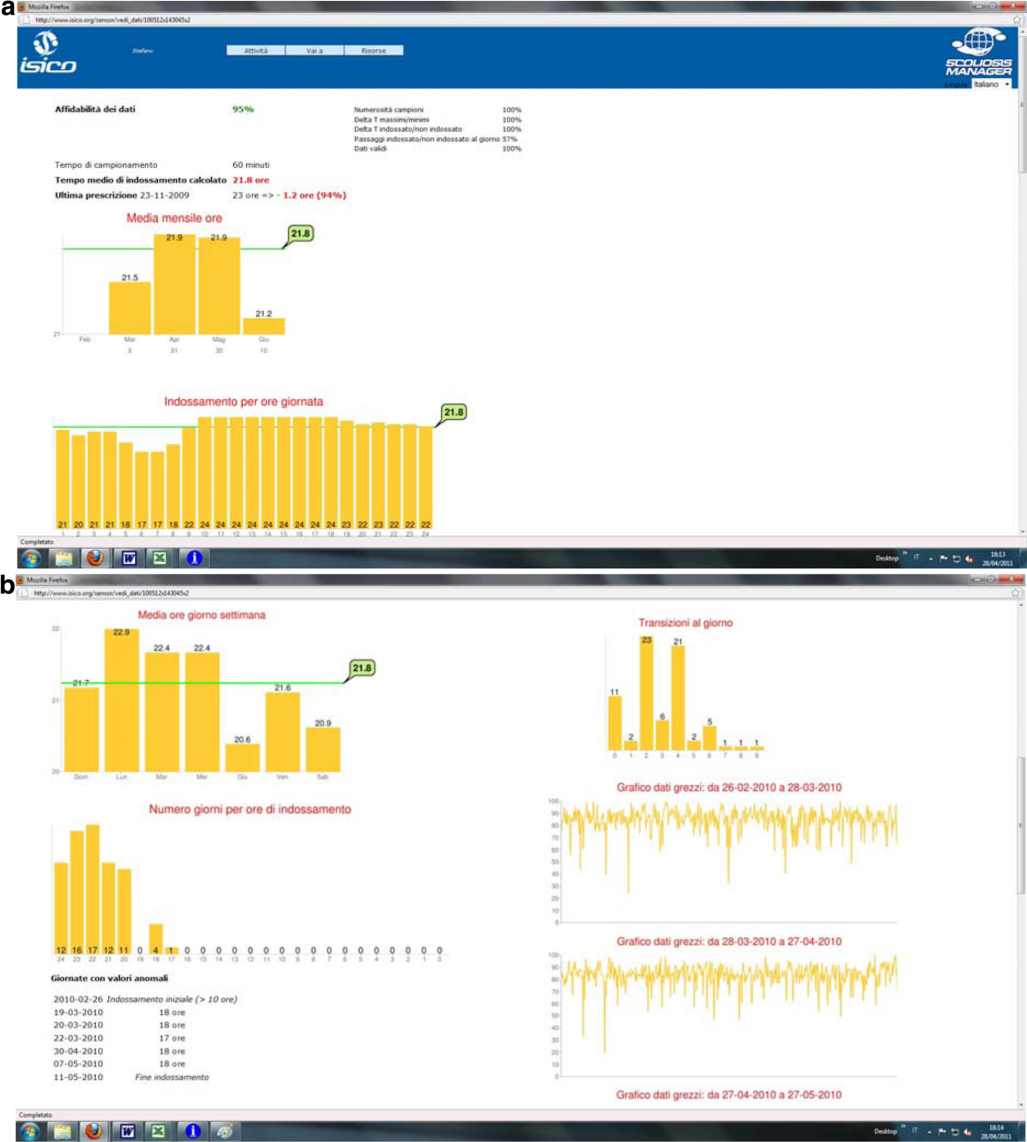
**Example of patient compliance data.** Screenshots of the specially developed software for everyday use (freely usable in Internet: http://www.scoliosismanager.org/thermobrace) with an example of patient compliance data. **a**: Reliability data, time of use, compliance (in red) and comparison with last prescription; graph of average use per month; graph of average use per hour of the day. **b**: on the left: graph of average use per day of the week; graph of number of days per daily hours of use; exceptions (i.e., day of use very different from the others). On the right: graph of daily transitions (i.e. brace on/off cycles) and raw data.

This specific software has been integrated with our clinical system (http://www.scoliosismanager.org). Today it allows clinicians to systematically access the following data:

Reliability of data (Figure 3a)

Average wearing time versus prescription (Figure 3a)

Graph of the average wearing time per month (Figure 3a)

Graph of the average wearing time per hour of the day (Figure 3a)

Graph of the average wearing time per day of the week (Figure 3b)

Graph of the number of days in which the brace was worn for a specific number of hours (Figure 3b))

Anomalous days (Figure 3b))

Graph of number of transitions brace on/brace off per day (Figure 3b)

Graph of raw data (Figure 3b)

### Reliability of thermobrace and temperature effects in different seasons

**Figure 4:**
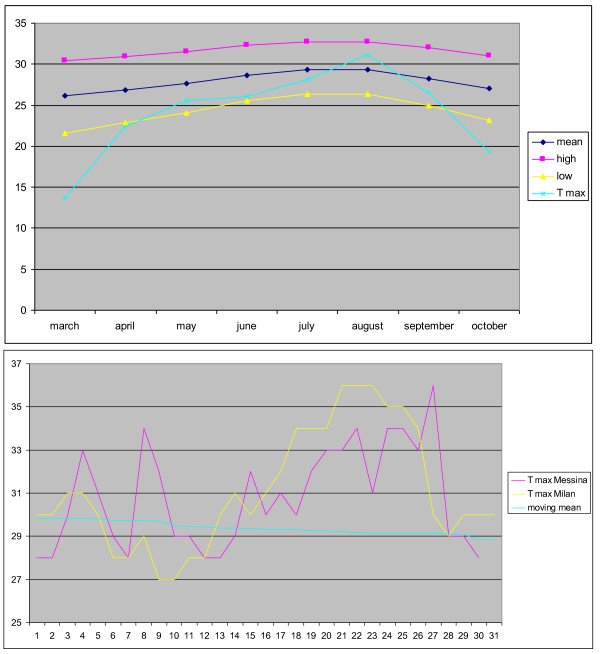
**Clime temperature and comparison among environmental temperature in the north and in the south of Italy.** The upper graph shows that the hottest period of the year was during the month of August, in the lower part the graph shows that the trendo of temeprature in Milan and in Messina were very similar.

Environmental clime and temperature didn’t seem to affect data recorded by the heat sensor. This is a very challenging question and, due to reviewers suggestions, we analyzed our data in the attempt to investigate this possibility. So in a wider group of 312 patients (obtained in February 2012, after the original study was performed) we collected the moving mean obtained by the TB, for the high and low threshold in each months of the year. We looked for the hottest period of the year and we compared the average temperature per hour of the day with the mean brace wearing time per hour of the day in the same month. Moreover, we compared the mean moving average obtained from the TB in a city of the North of Italy with one in the South, as well as the hottest temperatures in the same period in these two towns: the trend was similar. The Figure 4 A and B shows the mean temperature per months of the year and the comparison of temperatures in Milan (North) and in Messina (South).

In the hottest period of the year (August) the wearing threshold has been passed for 208 hours, the worst possible effect is that a patient with a prescription of 23 hours per day, after a monitoring period of six months, may show a wearing time of 23 hours per day, measured by Thermobrace, while the real wearing time being of 21.8 hours per day. This is the worst possible case and the overestimation is quite little.

An additional indirect analysis can be done by checking the changes in wearing rates between the months of August and the nearest months (July and September): on average in August (the hottest month of the year), the brace has been worn 28 minutes more than in July (this could be an overestimation effect of the external temperature), but also 1 hour and 20 minutes fewer than in September (and this is in contradiction, since in Italy in September the temperature is considerably reduced when compared to August). Moreover, we have to consider that during summer people goes on holiday and have journeys, these differences are not significant and cannot be justified only by the hot weather.

Finally, with respect to the external weather, braces are used mainly at home, and in Italy most of the houses have air conditioning, so greatly limiting this possible, theoretic overestimation effect.

### Compliance

We considered two different types of compliance:

Referred compliance: The ratio of brace wear time reported by the patient as to the prescribed brace regimen;

Real compliance: The ratio of brace wear time measured with the TB as to the prescribed brace regimen.

To calculate compliance, we considered the total amount of hours of brace-wearing from the brace check (TB mounting) to the first clinical follow-up (data discharge).

### Treatment

All patients have been treated with braces following the SPoRT principles [3,4,29-31]: Sforzesco, Sibilla or Lapadula. The acronym “SPoRT” means “Symmetric, Patient-oriented, Rigid, Three-dimensional, active” and has been defined because these braces are designed to be as much as possible bodily shaped and adherent (Figure 2) so as to be easily masked underneath clothing; moreover, these braces allow complete movement with the limbs so as to permit sports activities, which are frankly encouraged [32]. All the patients also performed exercises according to the SEAS principles [4,33-35].

The setting in which treatment has been proposed fulfilled all the requirements of the SOSORT Braced Patients Management Guidelines: answering to the specific questionnaire reported in the paper [15], Excellent Results have been obtained, with 43 out of 44 criteria respected; 1 being not applicable. Accordingly, the patients have been followed by a highly trained team focused on maximizing compliance and minimizing the Quality of Life and psychological impacts of brace treatment [36].

### Statistics

Because the normal distribution was almost never verified (Wilk-Shapiro test), we used median and 95% interval of confidence (IC95) to describe data, as well as non-parametric tests (Kruskal-Wallis, Wilcoxon and Kolmogorov-Smirnov). When the normal distribution was verified we used average, standard deviation and parametric tests (ANOVA and *t*-test). The statistical significance has been set at p < 0.05.

## Results

TB usage lasted an average of 5.5 ± 1.5 months, with no statistical differences between the groups. In the main group (first prescription of a brace) the brace had been prescribed 23 hours/day (h/d) (range 18–23); patients referred to wearing the brace for a median of 23 h/d (IC95 14.7-23) (100% referred compliance; IC95 70.7-100), but the measured wearing time was 21 h/d (IC95 11–23) with a 91.7% real compliance (IC95 56.6-100) (Table 3).

**Table 3:** Compliance and brace prescription in the two main groups studied

	**First brace prescription**		**Already in brace**	
	**Hours**	**%**	**Hours**	**%**
*Referred compliance before starting the study*				93.1
				(65.2-105.4)
*Brace prescription*	23		18	
	(18–23)		(15.9-23)	
*Actual referred compliance*	23	100	17	100
	(14.7-23)	(70.7-100)	(13.9-23)	(73.1-104.6)
*Measured compliance*	21	91.7	16.4	91.3
	(11–23)	(56.6-101.7)	(11.2-22.2)	(56.8-112.3)

Compliance and brace prescription in the two main groups studied. The reported data are median and 95% interval of confidence.

In the second group of patients already wearing a brace before the study, TB usage motivated an increase of referred compliance when compared to that before wearing the TB (even if not statistically significant), while both referred and measured compliance corresponded to those of the main group (Table 4).

**Table 4:** Everyday use of the brace versus prescription

	***Percentage of considered days***	
*At least 3 hours more*	13.5 (0–29.8)	
*1-2 hours more*	17.3 (0.9-53.6)	61.6 (4.3-99.6)
*Prescription*	17.7 (0.6-48.2)	
*1-2 hours less*	21.9 (2.3-53.9)	
*At least 3 hours less*	38.0 (4.3-99.6)	
*No wearing*	1.9 (0–6.6)	

Percentage of the days considered in which patients maintained the prescribed hours, or changed according to what reported in each line. In 56.2% of the days patients remained around 2 hours from what was prescribed, while non-wearing days during assessment period were 2.1% of the total. The reported data are median and 95% interval of confidence.

**Figure 5:**
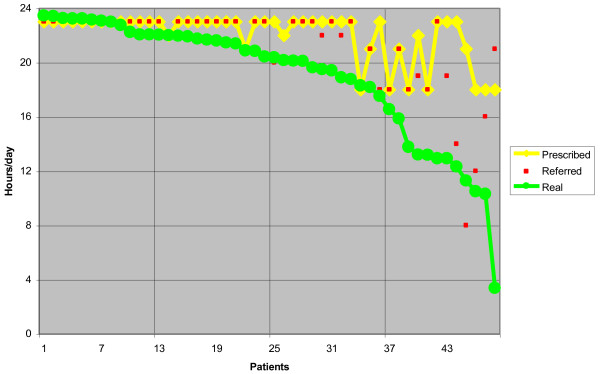
The real compliance was high, even if frequently overestimated by patients and their parents.

**Figure 6:**
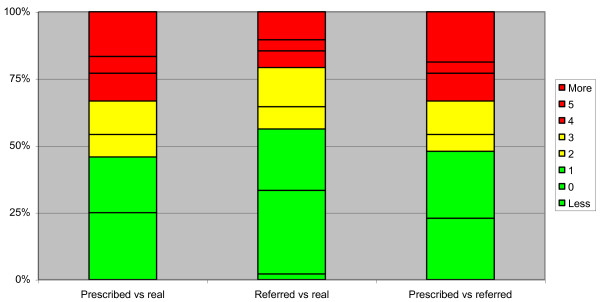
**Hours of difference from what was prescribed and what was referred by patients.** Graph reporting the hours of difference from what was prescribed and what was referred by patients. In green the best range (0–1 hours difference), in yellow an almost acceptable (2–3 hours difference), in red the not acceptable (4 or more hours of difference). Nearly 45% of patients remained in the range of 1 hour from what was prescribed and 55% based on what they referred.

Real compliance was high, despite being overestimated by patients and their parents (referred compliance) (Figure 5). Nearly 45% of patients remained in the range of 1 hour from what was prescribed, while the figure was 55% based on their own referred compliance (Figure 6); 60% of patients had at least 90% compliance. In 56.2% of the days the patients remained approximately 2 hours from what was prescribed, while there was a median of non-wearing days during the assessment period of 0 (IC95 0–12.95), which involved 29.2% of patients (8.3% had only 1 non-wearing day) (Table 3). We found no difference in compliance based on gender, while brace prescription had an influence: the more hours were prescribed, the higher the compliance was (94.8% in the group 23 h/d vs. 73.2% in the group 18 h/d) (P < 0.05) (Table 4).

**Figure 7:**
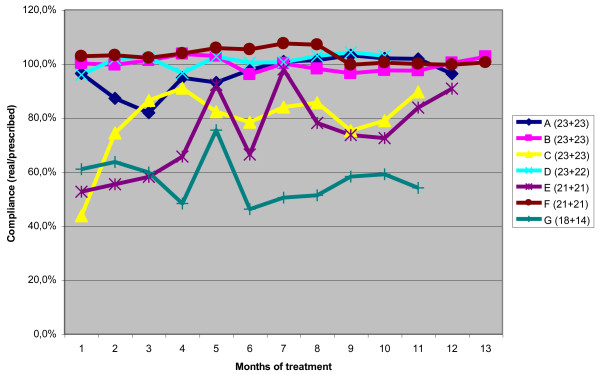
**Patients with more than two checks through Thermobrace.** Patients with more than two checks through Thermobrace (two clinical evaluations almost every 6 months): If some differences can be seen, there were no statistically significant differences during the months in the general population. Single patients tend to maintain the same compliance rate, with a few exceptions.

The maximum brace wear (92%) was reached during the night, while the minimum (64%) was in the afternoon and evening (Table 5). No statistically significant differences were observed during the months, even if in single patients some differences could be seen (Figure 7) (patients with more than one TB check). There were no differences between weekdays and weekends; on the contrary, it was possible to identify one best and one worst day of the week in 37.5% of patients (a difference of at least 1 hour from the average), and the difference of 1.9 hours between the two was statistically significant, as shown in Table 6.

**Table 5:** Results in the subgroups considered: gender and prescribed hours per day

		**Gender**		**Hours/day**		
		**Males**	**Females**	**23**	**21-22**	**18**
*Referred compliance*	*%*	100	100	100	100	100
		(63.5-100)	(81.9-100)	(81.1-100)	(52.2-100)	(73.3-111.7)
	*P*	NS		NS		
*Measured compliance*	*%*	89.6	93.6	94.8	86.5	73.2
		(55.7-101.8)	(55.8-101.8)	(56.2-101.8)	(55.6-99.7)	(28.4-98.7)
				P < 0.05		
	*P*	NS		NS		
				P < 0.05		

We found no difference in compliance for gender, while brace prescription had an influence: the more the hours prescribed, the higher the compliance. The reported data are median and 95% interval of confidence.

**Table 6:** Average wearing time in hours found with the Thermobrace

		
***Per month***	***1*^*st*^**	**21.5 (9.8-23.4)**
	***2*^*nd*^**	**21.3 (10.5-23.5**)
	***3*^*rd*^**	**20.7 (10.8-23.5)**
	***4*^*th*^**	**20.5 (12.1-23.5)**
	***P***	**NS**
*Per hour of the day*	*Night (0–7)*	7.7 (4.9-8)
	*Morning (8–13)*	5.4 (3.7-6)
	*Afternoon (14–19)*	4.6 (3.7-6)
	*Evening (20–23)*	3.2 (2.5-4)
	*P*	<0.05
*Per day of the week*	*Best day*	19.9 (12.3-23.5)
	*Worst day*	18.0 (9.6-23.1)
	*P*	<0.05
	*During the week*	20.6 (4.4-23.3)
	*Weekend*	20.5 (4.5-23.3)
	*P*	NS

Maximum brace wear is reached during the night. Forty-three percent of patients showed a best and a worst day of the week (a difference of at least 2 hours of brace-wearing), with a statistically significant difference. The reported data are median and 95% interval of confidence.

## Discussion

According to these results, as obtained using TB consecutively in an everyday clinical setting for more than 5 months, it is possible to achieve very good compliance (91.7%) even if it is lower than what is referred by patients. In this respect we cannot really know how much of what patients refer is driven by their own inaccurate perception or how much is due to deceptiveness. In our study the patients wore the brace for a median of 21 hours per day, with 45% who remained at a maximum of 1 hour from what was prescribed. While there was no difference based on gender, interestingly the patients with the highest prescription (23 h/d) used the brace more than those with the lowest (18 h/d).

**Figure 8:**
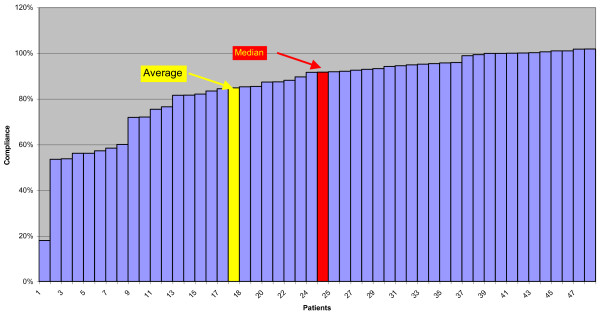
**Histogram of the compliance percentages.** This graph illustrate the compliance of each patient. Since we find a not normal distribution and data were positively skewed, in statistical terms the median represents more correctly than the average our results.

Since it is very hard to compare results from compliance monitors, there are some more points of clarification worthy of mention: it is unusual to report on median points of data rather than average. This is the correct statistical representation of our data because of the abnormal distribution, which is represented in Figure 8; in fact in cases like this the use of average and standard deviation leads to misunderstanding of data and in this specific case to an important underestimation (−8%) of the wearing time.

We are aware of the limits of this study and we know that comparison of our results with previous published data is quite uneasy, because few studies have been published and they used different types of compliance monitors; moreover, each research had different aim and modalities; anyway, results of previous studies are shown in Table 7, with the only aim to arise some interesting issues for further considerations.

**Table 7:** Literature compliance results

***Author***	**Aim**	**Patients**	**Compliance results**
*Vandal 1999*[20]	To report the observed difference between two measures of compliance: interview and a compliometer.	40 female patients with AIS aged 10–16 years	Compliance measured with interview: 82.5%, compliometer: average compliance 33%
*Havey 2002*[21]	To develop and test the reliability of a device (pressure sensor) for the objective measurement of spinal orthosis wearing time.	9 normal volunteers	Compliance with brace- wearing can be accurately measured by an electronic device embedded in the orthosis.
*Nicholson 2003*[12]	To develop instrumentation for discrete, reliable and objective measurement of brace usage patterns.	10 female pts with AIS (mean age 15)	Effective compliance: 65% (range 8%-90%) patients generally overestimated their time in brace.
*Takemitsu 2004*[23]	To evaluate objectively idiopathic scoliosis patients’ compliance with Wilmington brace treatment.	61 pts with AIS (mean age 12 range 6–16 years)	Average compliance was 75%, age related.
*Rahman 2005*[22]	To evaluate the efficacy of brace treatment prospectively using an objective measure of compliance.	34 pts average age 12 (range 10–16 years)	The compliance rate of the group whose curvatures did not progress was 85 + −18,5%; that of the group whose curvatures progressed was 62 + −24,3%.
*Helfenstein 2006*[24]	To establish new technical methods for the objective measurement of brace usage without patients’ involvement.	9 female patients with AIS	68% range 19%- 97%. No patient reached the recommended 23-hour bracing.
*Morton 2008*[19]	Testing prediction and estimation of adherence compared to the objective measures of compliance.	124 pts with AIS	Actual average of adherence was 47%. Physicians, orthotists, parents and patients respectively overestimated brace wear as 64%, 66%, 72% and 75%.
*Rahman 2010*[37]	To demonstrate the efficacy of using a new electronic brace compliance monitor.	10 AIS pts in Wilmington brace treatment	Patient compliance 78%. The cricket is a reliable accurate and sensitive device to determine compliance.
*Katz 2010*[26]	To measure accurately the number of hours of brace to determine if increased wear correlate with lack of progression.	100 pts in Boston brace therapy for AIS	the total number of hours of brace wear in patients who didn’t undergo surgery had a mean compliance of 42.4% a mean compliance of 24.4% in the group of patients needing for surgical treatment.
*Muller 2011*[27]	To objectify the impact of spinal bracing on daily step activity in AIS and adolescent kyphosis patients receiving conservative treatment with CTM brace and a kyphosis brace.	38 AIS patients and 10 AK patients using an ankle monitor for recording gait cycles and a temperature sensor to determine brace wear time.	The overall compliance rate was 72.7 + − 27.6%, based on the 23-hour recommended wearing time.

Literature compliance results. AIS: Adolescent Idiopathic Scoliosis.

Our data suggest us that our patients show a higher compliance to bracing, if compared to what previously reported:

The best real average compliance previously reported was 78% [22], other authors reported different ranges of compliance with exceptions down to 33% [20] and 47% [19]. In a couple of study a maximum of 90% [12] and 97% [24] have been reported, while the median obtained in this study was 91.7%;

It has been reported that no patients reached the recommended 23 h/d [24], and that 18 h/d was the maximum [26]: in our study 23 h/d was the median of real brace-wearing.

It has also been stated that patients with the prescription of 23 hours per day didn’t wear the brace for more hours than those with 16 hours of prescription [26], while in this study the best users were in the 23 h/d group;

Overestimation of real compliance has been reported, [12,19,20] which also corresponds to our findings.

It would be very interesting to understand what kind of factors could affect results and what elements could determine the differences obtained in all studies: the management of patients and the type of brace could be the two main items in our opinion.

Management of patients has received significant attention in the literature only recently, when everyday clinical experience made the experts of conservative treatment of SOSORT to develop some criteria to be respected during treatment [15,16]. In fact, these same experts did not find common pathways in brace construction [29,38-43], but only in the way they managed patients, so driving presumably their adherence to treatment [15,16]. In our everyday setting, the team construction is reality, and we devote a lot of efforts to cognitive behavioral techniques to increase compliance (and patients’ quality of life). From our perspective, this can certainly explain the results we found [44].

Together with management could come the type of brace. In fact, while the number of hours of prescription and the construction of the brace certainly affect, on one side, the final results, due to the specific quality of the specific orthosis [38-40,42], they can also influence adherence to treatment due to visibility, symmetricality and other factors [36,41,44]. Comparing our results to the compliance previously shown, we can imagine that the braces used in the present setting influenced the results; in fact, they belong to the SPoRT family (Symmetric, Patient-oriented, Rigid, Three-dimensional, active) [29] and, due to their external symmetry, which allows them to be easily masked beneath clothing, could help patients and contribute to compliance.

Nevertheless we have also to consider that in three out of the 10 papers considered in Table 7, the brace type is defined, Willmington brace [22,37] and Boston [26]. Both braces are symmetric and low visible. Contrary, in some of the papers reporting good end results [6,8,13], which would not be possible with bad compliance (not compliant patients were not excluded), the used brace was the Chêneau type brace, which is, by definition, an asymmetric brace producing usually postural over-correction. This demonstrate how some teams using Chêneau type braces are also able to achieve high compliance in their patients, no matter how asymmetric and visible is the brace. A possible conclusion is that, no matter the type of brace, it will produce a certain amount of physical and/or functional discomfort, which will produce secondarily ‘emotional discomfort’. Thus, try to make the brace more comfortable from the physical and functional point of view is a valid strategy. However, ‘emotionally discomfort’ is probably also primarily produced by other different factors, acting even before the patient is able to recognize any ‘physical and/or functional’ distress. While this, let us call it, primary ‘emotional discomfort’ persists, patients will find always a reason for a low compliance, complaining about physical and/or functional discomfort. Once ‘emotional discomfort’ decreases (different strategies can be used to capture emotionally patients and their parents), ‘physical and functional discomfort’ can be at the same time reduced and overcame, improving compliance. Thus, team approach can make theoretically any brace type to be wearable or not wearable. All these considerations leads to a possible conclusion, that the type of brace can matter, but the correct management of patients matter much more [15,44].

The difference reported in the literature in regard to compliance with bracing appears too similar to the differences found and reported in terms of the efficacy of bracing not to be correlated in some way. In fact, while previous studies demonstrated the efficacy of the conservative treatment of adolescent idiopathic scoliosis [1,2,4,5,8,9,45], others did not [11,46]. It is clearly possible that the difference is found in compliance: without an objective measurement of it, all the negative results remain questionable, since it is not possible to know what determined them. Compliance to treatment therefore represents one of the main conditions for the success of brace therapy: An orthosis cannot be effective unless it is both accepted and worn.

There are many reasons for which it is difficult to wear a brace: first of all, we are talking about teenagers, and it is difficult for a teenager to evaluate the long-term impact of non-compliant behavior [47]. Braces are often perceived as a threat to body image; and braces are visible to the others, has a negative impact on the perception of themselves in adolescence [16,47-51]. The relationship with the adolescent entourage and the attitude of close friends and relatives are particularly significant to adolescents. It has been demonstrated that the lack of symptoms associated with scoliosis and treatment-induced pain are detrimental to compliance [44,52]. If we think that a diabetic teenager is more likely to adhere to treatment than a teenager affected by scoliosis, everything becomes clearer.

The data was downloaded at each follow-up visit--usually every six months--so the average monitoring period was sufficiently long compared to some of the previous studies (but reduced when compared to others) [26]. For a small number of patients it was possible to collect more than one download: The data wasn’t sufficient to define a trend, but it seems that adherence to the wearing recommendation didn’t decline over time, as some might suppose. In that respect, also the management of patients followed in our institute, where with growth the hours of wear are progressively decreased [3,29,36], could help to maintain compliance over time (if not increase it). In this small group of patients we found that compliance didn’t change during treatment (Figure 7): Patients with low compliance remained so, and patients with compliance of 90% or more maintained these levels of adherence.

The possible limitations of the present study include the awareness of the patients of the presence of the TB, which might have affected patients’ compliance: nevertheless, if one thinks of what wearing plastic means, this seems the less important of the problems. In fact, we had not-compliant patients, even in front of the TB objective report. In any case, with this study we wanted also to test the use of the TB in routine clinical activity. Because of this, and because of ethical issues, patients and family had to be informed about the use of this device and had to accept it. Another limit is the possible selection bias, since not all patients have been included due to the experimental start of everyday clinical use. This should be overcome in the future with stricter studies.

Other limits of this study are the heterogeneous sample of patients analized and the little average period of monitoring (5.5 months). This study would be significantly strengthened by removing the subjects that were selectively enrolled after treatment had already begun: nevertheless, this was done in all main analysis.

Someone should advocate that also the quality of brace wearing is important: in fact, wearing a spinal orthosis only does not mean giving an effective treatment to the patients with AIS, until the orthosis prescribed tightness is maintained. Future research should question not only quantity but also real quality of treatment, by using also biomechanical monitor measuring forces produced within the brace.

Making this considerations we must remember that scoliosis treament best results are done of different elements: exercises, braces, team approach … So quality should be represented by end of growth results as the optimal interaction of all these elements.

### Comments on the everyday clinical use of thermobrace

Muller [53] in a recent study questioned the use of a TB in the everyday clinical practice, concluding that it is not to be recommended because patients might feel wary and the mutual trust needed for the confidential patient-doctor relationship might be negatively affected by the use of objective checking tools. According to these premises, and due to the same fear, we were very cautious at the beginning: The TB was presented to patients and family as a useful device with which to optimize the efficacy of therapy, explaining the importance of wearing a brace and of knowing the real wearing time to ensure adequate therapy with respect to the results actually obtained. When receiving patients, all the particulars offered by the software allowed a deepening of the real everyday use, making it possible, for example, to check for less compliant days of the week or for specific periods of not wearing, for daily transitions (brace on/off cycle) and so on. Consequently, we discovered that, when well used, the TB strengthens the patient-physician relationship through a frank discussion of real data, avoiding guesses that are sometimes totally wrong. Those guesses, if wrong, can really undermine a good relationship. Our experience has been that the use of the TB can contribute to the good results of therapy and become an integral part of the treatment.

Given the objective measurement of compliance, a reliable profile of compliers and non-compliers can be established so as to allow tailored treatment protocols and educational and supportive efforts to improve compliance. The use of the TB in the group of patients who had already started brace therapy had, as its main objective, the goal of facilitating the physician’s clinical choices: When a brace is not correctly used it is very difficult to dose the therapy adequately. In this group of patients, probably TB has been better accepted by parents than patients. In any case, the use of the TB didn’t have a negative effect on their compliance; on the contrary, after the use of the TB the referred compliance increased.

### Reliability of thermobrace

We developed an algorithm to cope with the seasonal changes of temperature. However, we must consider that the device may not be applicable to the regions with hot weather, so we did a further analysis of the collected data looking for the different regions of our country: the comparison didn’t show significant differences.

We can’t deny that there is the possibility that in the really worst case the hot weather could allow an overestimation (although little) of real compliance, but it is a very rare event and the fact that we use always statistically processed data decreases even more this risk. So the problem potentially exists, and should be better quantified with experimental studies in the future; on the other hand, at our latitudes (continental climate) the phenomenon appear for a limited period of the year, such that the influence on the data does not appear to affect results. The distribution of air conditioners and the fact that during the hottest hour of the day people in general, and patients wearing braces even more than the others, prefer to stay at home or in cool places, where the temperature is on average lower than outside, obviously contribute to limit this event.

## Conclusion

Compliance is due neither to the type of treatment nor to the patient alone. The SOSORT criteria for bracing clearly state the importance of the treatment team in this respect [15,16]. This was the first study to use a TB in a setting that was respectful of the SOSORT criteria, and shows a higher compliance to bracing than what was previously reported.

It is possible not only to make the patient wear a brace, even with a full-time prescription, but it is also possible to have referred compliance that is close to the real compliance. Although the contribute of an electronic device to measure actual compliance is fundamental for research purposes, in the everyday clinical setting the TB offers valuable insight by which to increase compliance even further and make treatment rely on real data. From that perspective the TB has become an integral part of the treatment. According to our experience, an electronic device does not negatively affect the relationship between the patient and the doctor.

This was a preliminary study, the results emphasize the importance of further research with greater and homogenous samples of consecutive patients. Longer monitoring periods are needed, to better understand the efficacy of brace treatment and to point out all factors involved in the adolescent idiopathic scoliosis treatment. The opportunity to measure real compliance by monitoring all patients until the end of therapy, will allow a deeper analysis about the real efficacy of brace treatment.

## Competing interests

The authors declare that they have no competing interests.

## Authors' contributions

SD and SN collected data and drafted the text and figures; FZ revised and accepted it, and contributed to the text and figures. All authors read and accepted the final version of the manuscript
